# CXCL9, IL2RB, and SPP1, potential diagnostic biomarkers in the co-morbidity pattern of atherosclerosis and non-alcoholic steatohepatitis

**DOI:** 10.1038/s41598-024-66287-4

**Published:** 2024-07-16

**Authors:** Xize Wu, Changbin Yuan, Jiaxiang Pan, Yi Zhou, Xue Pan, Jian Kang, Lihong Ren, Lihong Gong, Yue Li

**Affiliations:** 1 Liaoning University of Traditional Chinese Medicine, No. 79 Chongshan East Road, Huanggu District, Shenyang, 110847 Liaoning China; 2 Nantong Hospital of Traditional Chinese Medicine, Nantong Hospital Affiliated to Nanjing University of Chinese Medicine, Nantong, 226000 Jiangsu China; 3 The Affiliated Hospital of Liaoning University of Traditional Chinese Medicine, Shenyang, 110032 Liaoning China; 4 Liaoning Provincial Key Laboratory of TCM Geriatric Cardio-Cerebrovascular Diseases, Shenyang, 110847 Liaoning China; 5 Dazhou Vocational College of Chinese Medicine, Dazhou, 635000 Sichuan China

**Keywords:** Bioinformatics, Differentially expressed genes, Weighted gene co-expression network analysis, Immune infiltration microenvironment, Cardiovascular diseases, Computational biology and bioinformatics, Biomarkers, Diseases

## Abstract

Non-alcoholic steatohepatitis (NASH) is a hepatocyte inflammation based on hepatocellular steatosis, yet there is no effective drug treatment. Atherosclerosis (AS) is caused by lipid deposition in the endothelium, which can lead to various cardiovascular diseases. NASH and AS share common risk factors, and NASH can also elevate the risk of AS, causing a higher morbidity and mortality rate for atherosclerotic heart disease. Therefore, timely detection and diagnosis of NASH and AS are particularly important. In this study, differential gene expression analysis and weighted gene co-expression network analysis were performed on the AS (GSE100927) and NASH (GSE89632) datasets to obtain common crosstalk genes, respectively. Then, candidate Hub genes were screened using four topological algorithms and externally validated in the GSE43292 and GSE63067 datasets to obtain Hub genes. Furthermore, immune infiltration analysis and gene set variation analysis were performed on the Hub genes to explore the underlying mechanisms. The DGIbd database was used to screen candidate drugs for AS and NASH. Finally, a NASH model was constructed using free fatty acid-induced human L02 cells, an AS model was constructed using lipopolysaccharide-induced HUVECs, and a co-morbidity model was constructed using L02 cells and HUVECs to verify Hub gene expression. The result showed that a total of 113 genes common to both AS and NASH were identified as crosstalk genes, and enrichment analysis indicated that these genes were mainly involved in the regulation of immune and metabolism-related pathways. 28 candidate Hub genes were screened according to four topological algorithms, and CXCL9, IL2RB, and SPP1 were identified as Hub genes after in vitro experiments and external dataset validation. The ROC curves and SVM modeling demonstrated the good diagnostic efficacy of these three Hub genes. In addition, the Hub genes are strongly associated with immune cell infiltration, especially macrophages and γ–δ T cell infiltration. Finally, five potential therapeutic drugs were identified. has-miR-185 and hsa-miR-335 were closely related to AS and NASH. This study demonstrates that CXCL9, IL2RB, and SPP1 may serve as potential biomarkers for the diagnosis of the co-morbidity patterns of AS and NASH and as potential targets for drug therapy.

## Introduction

Nonalcoholic fatty liver disease (NAFLD) refers to the development of hepatic steatosis in the absence of secondary causes such as excessive alcohol consumption. It encompasses conditions including simple steatohepatitis, nonalcoholic steatohepatitis (NASH), and liver cirrhosis, with NASH being the inflammatory subtype of NAFLD^1^. It is estimated that NAFLD affects approximately 25% of the global population, with NASH accounting for 59.10% of cases within the NAFLD patient group, a number that is increasing year by year^2^. NASH causes more severe liver damage than simple steatohepatitis and is more likely to result in liver fibrosis, further progression to cirrhosis or liver cancer, and even death^3,4^. NASH has become the second-leading cause of liver transplantation; however, there are no drugs approved by the Food and Drug Administration (FDA), and treatment options remain very limited^5,6^.

Atherosclerosis (AS) is a chronic inflammatory pathological change that occurs in the vascular wall and is characterized by lipid deposition and immune cell infiltration. This condition can result in various cardiovascular diseases (CVDs), such as ischemic heart disease and stroke^7^. CVDs is the leading cause of morbidity and mortality worldwide, with an increasing trend year by year^8^. AS serves as the pathological basis of CVDs and has been the subject of research by the SCAPIS project in Sweden. Among 25,182 people with no known coronary heart disease, 42.1% showed evidence of atherosclerosis on coronary computed tomography angiography^9^. The current treatment of AS is dominated by statins for lipid lowering; however, there is still a significant residual risk of atherosclerotic cardiovascular disease even under optimal statin therapy^10^.

Chronic diseases are intricately linked, and some often occur in conjunction with each other. AS and NASH are common clinical chronic diseases. A high-fat diet disrupts metabolic rhythms, affecting and altering communication between tissues or organs, which in turn weakens synergistic metabolic associations between organs and causes abnormalities in biometabolism. This disruption contributes to the development of co-morbidities of AS and NASH^11,12^. Recent clinical studies have shown that NASH increases the risk of atherosclerotic cardiovascular disease; patients with NAFLD have twice the risk of cardiovascular disease, and individuals with NAFLD/NASH are twice as likely to die from cardiovascular disease as from liver disease^13–15^. Therefore, studies on co-morbidity patterns can help to understand the characteristics of chronic disease co-morbidities and guide clinical diagnosis, treatment, management, and etiological research into these co-morbidities.

However, the current state of co-morbidity presents unprecedented challenges, as disease-specific diagnostic and therapeutic protocols based on single-disease guidelines may not be suitable for patients with co-morbidities. Moreover, research into the diagnosis, treatment, and underlying mechanisms of co-morbidities is crucial for medical advancement, and the development of coping strategies is particularly important. The guidelines acknowledge that invasive liver biopsy is the gold standard for diagnosing NASH, but its limitations, such as sampling bias, poor acceptability, and serious complications including mortality, bleeding, and pain, have highlighted an urgent need for noninvasive methods to avoid biopsy in the diagnosis of NAFLD^16^. Biomarkers are ideally suited for diagnosing co-morbidities of AS and NASH, which enable the non-invasive detection of these conditions at their onset and are instrumental in tracking the progression of the diseases and monitoring the effectiveness of treatments. Moreover, biomarkers offer insights into the underlying mechanisms of these co-morbidities, which is crucial for developing targeted therapies. This study offers diagnostic methods and potential therapeutic targets for the co-morbidity of NASH and AS, plays a key role in advancing the treatment of these diseases, and provides new ideas for the development of new drugs.

## Materials and methods

### Bioinformatics analysis

#### Subjects and dataset acquisition

**Figure 1:**
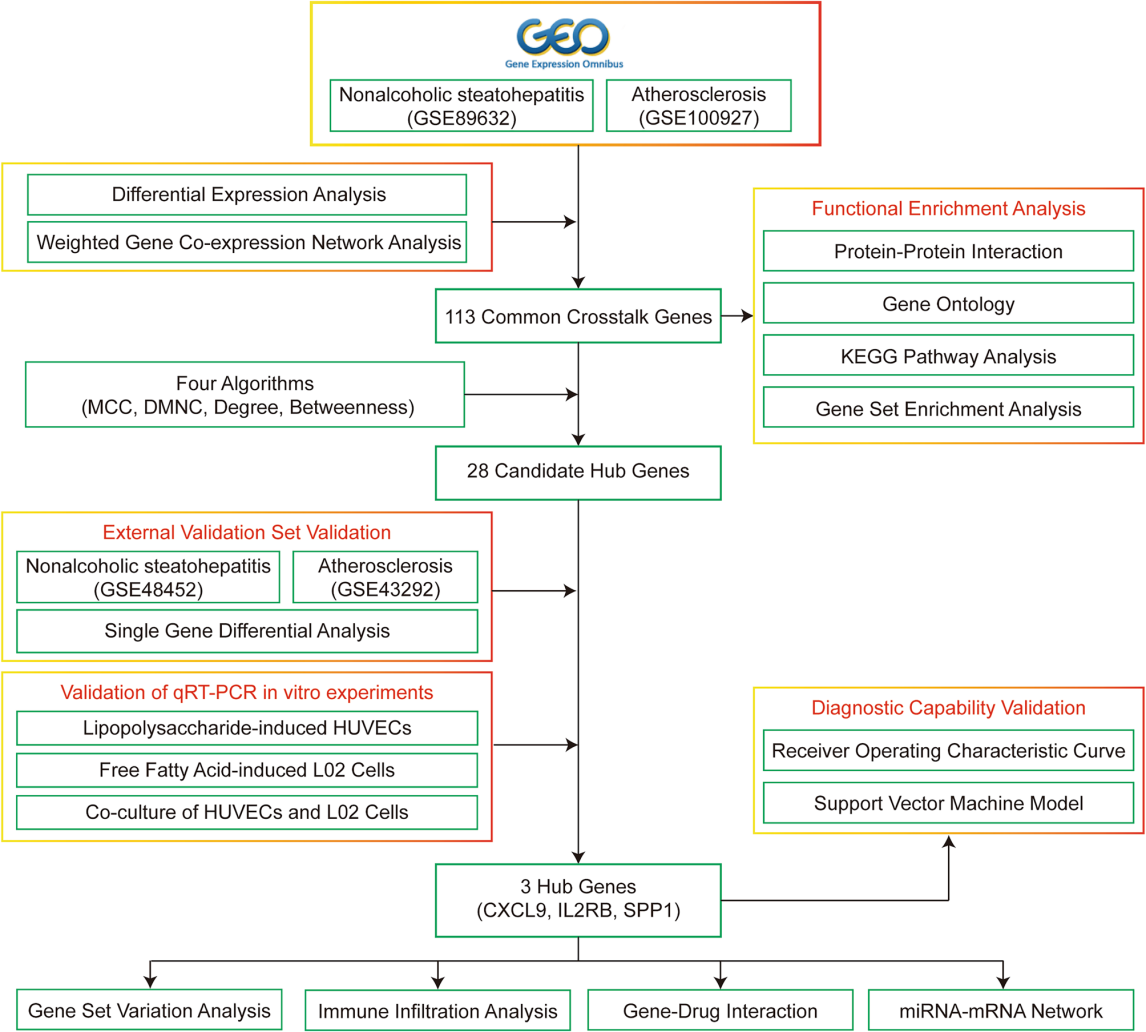
Flow chart of this study.

The entire study process is illustrated in Fig. 1, and the study code is detailed in Supplementary Material A. First, gene expression profiles (GSE100927 and GSE89632) were retrieved from the Gene Expression Omnibus (GEO, https://www.ncbi.nlm.nih.gov/geo/) database searching under the keywords “atherosclerosis” and “nonalcoholic steatohepatitis,” with the species filter set to “*Homo* *sapiens*.” The datasets were selected based on the following criteria: (1) the presence of both control and disease groups; (2) specific test tissues for AS (artery) and NASH (liver); (3) a minimum sample size of 10 cases per group. The AS-related dataset, GSE100927, includes mRNA sequencing data from 35 healthy control arteries and 69 arteries from atherosclerotic patients, totaling 104 samples^17^. The NASH-related dataset, GSE89632, includes mRNA sequencing data from 24 healthy liver tissues and 19 liver tissues from NASH patients, totaling 43 samples^18^.

#### Identification of common crosstalk genes in AS and NASH

The common crosstalk genes of AS and NASH were screened using the “limma” and “WGCNA” R packages in the R software^19,20^. The differentially expressed genes (DEGs) for AS-GSE100927 were initially identified using the “limma” R package, comparing the disease and control groups with a significance threshold of *P* < 0.05 and |logFC| > 0.5 for DEG selection. Subsequently, the samples were clustered using the “WGCNA” R package, outliers were removed, and co-expression networks were constructed from the gene expression matrix of the remaining samples. Appropriate soft thresholds were determined, gene modules were identified, and the most relevant gene modules were selected. The crosstalk genes for AS were then obtained by intersecting the genes from the difference analysis with those from the Weighted Gene Co-expression Network Analysis (WGCNA). The crosstalk genes for NASH-GSE89632 were identified in a similar manner. Finally, the common crosstalk genes between AS and NASH were determined by intersecting the crosstalk genes of NASH with AS, ensuring that the genes expressed in the same direction (either upregulated or downregulated).

#### Gene Ontology (GO) and Kyoto Encyclopedia of Genes and Genomes (KEGG) enrichment analysis

The common crosstalk genes were imported into the David database (https://david.abcc.ncifcrf.gov/) for GO and KEGG analysis, with *P* < 0.05 set as the screening condition^21,22^.

#### Construction of protein–protein interaction (PPI) network and screening of candidate Hub genes

The common crosstalk genes were imported into the STRING database (https://www.string-db.org/) to construct a PPI network, and “*Homo* *sapiens*” was selected as a species, with a “score” of ≥ 0.4^23^. The topology analysis of PPI networks was performed using the Cytoscape plugin “CytoHubba,” and four algorithms (MCC, DMNC, Degree, and Betweenness) were selected to screen candidate Hub genes^24^.

#### Validation of Hub gene expression

The expression levels of the candidate Hub genes were verified in the datasets GSE43292 for AS and GSE48452 for NASH. GSE43292 contained 32 carotid tissues from AS patients and 32 control tissues^25^, while GSE48452 contained 17 liver tissues from NASH patients and 12 control liver tissues^26^. The Wilcoxon test was used to compare the two datasets, with *P* < 0.05 considered significant.

#### Receiver operating characteristic (ROC) curves

ROC curves for the Hub genes were generated using the “pROC” R package to evaluate the diagnostic efficacy of individual Hub genes^27^.

#### Support vector machine (SVM)

SVM models were constructed in the training and validation sets using the “e1071” R package (https://cran.r-project.org/web/packages/e1071/index.html), and then the ROC curves were used to evaluate the joint diagnosis of Hub genes' performance.

#### Immune infiltration analysis

The CIBERSORT deconvolution algorithm was applied to gene microarray data to quantify the infiltration of 22 immune cells^28^. Differences between groups were compared using the Wilcoxon test, and the results were visualized with the “vioplot” R package^29^. Spearman correlation analysis was then conducted to explore the relationships between Hub genes and immune cells.

#### Gene set enrichment analysis (GSEA)

The “GSEA” R package was utilized to investigate the pathways related to hub genes and to calculate the correlation between hub genes and other genes^30^. Genes were sorted by their correlation values in descending order, and this sorted gene set was used for enrichment analysis. The KEGG signaling pathway set, referred to as a “predefined set,” was assessed for its enrichment within the gene set.

#### Gene set variation analysis (GSVA)

The “GSVA” R package was employed to perform a GSVA enrichment analysis for each model gene, and a significant change was considered to have occurred if the |t| value of the GSVA score's t statistic exceeded two^31^.

#### Hub gene–drug interaction

Hub genes were imported into the DGIbd database (https://dgidb.org/) to identify drug candidates and screen for FDA-approved drugs.

#### Construction of common miRNA–mRNA network

The miRTarbase (http://mirtarbase.mbc.nctu.edu.tw/php/index.php) is an experimentally validated miRNA target interaction database^32^. MiRNAs targeting Hub genes were obtained from the miRTarbase database; four bioinformatics tools were then utilized to predict miRNA target genes, including the TargetScan database (http://www.targetscan.org/vert_72/), the TarBase database (https://dianalab.e-ce.uth.gr/tarbasev9), the miRmap database (https://mirmap.ezlab.org/), and the miRDB database (https://mirdb.org/), where miRNAs of Hub genes were selected by at least three tools at the time of prediction^33^.

#### Statistical analysis

All bioinformatics statistical analyses were conducted using R software, with *P* < 0.05 considered significant.

### Cellular experimental validation of Hub gene

#### Reagents and instruments

Experimental cell: Human umbilical vein endothelial cells (HUVECs) (Procell, Cat.CL-0675); Human normal liver L02 cells (iCell, Cat.h054).

The following reagents were used: Lipopolysaccharide (LPS) (Solarbio, Cat.L8880); Oleic acid (Solarbio, Cat.SC9320); Palmitic acid (Solarbio, Cat.SP8060); Fetal bovine serum (Biosharp, Cat.BL205A); Trypsin–EDTA Solution (Biosharp, Cat.BL512A); RPMI-1640 cell culture medium (gibco, Cat.C11875500BT); Penicillin/Streptomycin (Biosharp, Cat.BL505A); Oil red O (ORO) staining Kit (Solarbio, Cat.G1262); Paraformaldehyde, 4% (Solarbio, Cat.P1110); Cell counting kit-8 (Solarbio, Cat.CA1210); Serum-free cell freezing medlum (Biosharp, Cat.BL203B); RNA extraction solution (Servicebio, Cat.G3013); Phosphate-Buffered Saline (PBS) (Servicebio, Cat.G4202); Chloroform substitute (Servicebio, Cat.G3014); RNA lysate (Servicebio, Cat.G3029); Water Nuclease-Free (Servicebio, Cat.G4700); SweScript All-in-One RT SuperMix for qPCR (One-Step gDNA Remover) (Servicebio, Cat.G3337); 2×Universal Blue SYBR Green qPCR Master Mix (Servicebio, Cat.G3326); isopropanol (Sinopharm, Cat.80109218); Anhydrous ethanol (Sinopharm, Cat.10009218).

The following instruments were used: Inverted microscope (Nikon, Eclipse Ci); Microplate reader (Tecan, Spark 10M); Vortex mixer (Servicebio, SMV-3500); Sealing instrument (Servicebio, FS-A20); Microplate centrifuge (Servicebio, SMP-2); High-speed frozen microcentrifuge (DragonLab, D3024R); Fluorescent quantitative PCR instrument (Bio-rad, CFX Connect); PCR instrument (Eastwin, ETC811).

#### Cell culture

The HUVECs and L02 cells were cultured in RPMI-1640 cell culture medium supplemented with 10% fetal bovine serum and 1% penicillin/streptomycin, respectively, and incubated at 37 °C in humidified 5% CO2. The cells were used from passages 3–8^34^.

L02 cells and HUVECs were divided into 4 groups: (1) Control L02 group (CL): L02 cells are cultured normally without any treatment; (2) Model L02 group (ML): L02 cells were treated with free fatty acid (FFA) (2.0 mmol/L) (oleic acid = 2 mmol/L, palmitic acid = 1 mmol/L) for 24 h; (3) Control HUVECs group (CH): HUVECs are cultured normally without any treatment; (4) Model HUVECs group (MH): HUVECs were treated with LPS (1.0 μg/mL) for 24 h; (5) The control L02 cell and the control HUVECs cell co-culture group (CL + CH): L02 and HUVECs were co-cultured normally for 24 h without any treatment; (6) The control L02 cell and the model HUVECs cell co-culture group (CL + MH): L02 cells were cultured untreated for 24 h, co-cultured with HUVECs after changing the culture medium, and treated with LPS for 24 h; (7) The model L02 cell and the control HUVECs cell co-culture group (ML + CH): L02 cells were treated with FFA for 24 h and co-cultured with HUVECs for 24 h after changing the culture medium; (8) The model L02 cell and the model HUVECs cell co-culture group (ML + MH): L02 cells were treated with FFA for 24 h, HUVECs were treated with LPS for 24 h, and then co-cultured with L02 cell supernatant.

#### ORO staining

Cells were fixed in 4% paraformaldehyde for 30 min and then rinsed three times with PBS. Subsequently, the cells were stained with ORO for 1 h at room temperature.

#### Cell viability assay

Cells were cultured in 96-well plates at a density of 2 × 10^5^ per well. After treatment with different interventions for 24 h, 10 µL of CCK-8 reagent was added to each well and incubated at 37 °C for 1 h. The absorbance was measured at 450 nm using a microplate reader.

#### Quantitative real-time polymerase chain reaction (qRT-PCR)

Total RNA was extracted from cell lines using Trizol total RNA isolation reagent (Invitrogen) following the manufacturer’s specifications and treated with Turbo DNase (Ambion). cDNA was synthesized from 0.5 mg of total RNA using random hexamers with the TaqMan cDNA Reverse Transcription Kit (Applied Biosystems). Primers were designed using Primer Express v3.0 software, and real-time PCR was performed using SYBR Select Master Mix (Applied Bio-Systems) (Table 1). All reactions were carried out on the 7500 Fast Real-Time PCR System (Applied Biosystem). The average of three independent analyses for each gene and sample was calculated using the DD threshold cycle (Ct) method and normalized to the endogenous reference control gene β-actin. The above primers were synthesized by Sangon Biotech (Shanghai) Co., Ltd (https://www.sangon.com/).

**Table 1:** A list of the primers used in the qRT-PCR.

Gene	Primer sequence (5′–3′)
β-actin	Forward: CACCCAGCACAATGAAGATCAAGAT
	Reverse: CCAGTTTTTAAATCCTGAGTCAAGC
CXCL9	Forward: CATCTTGCTGGTTCTGATTGGAGTG
	Reverse: ATAGTCCCTTGGTTGGTGCTGATG
IL2RB	Forward: GAAATCTCCCAAGCCTCCCACTAC
	Reverse: ACCCGCACCTGAAACTCATACTG
OAS1	Forward: GGCGAGTTCTCCACCTGCTTC
	Reverse: GTGCTTGACTAGGCGGATGAGG
OAS2	Forward: TTCCAGGATCAGAAGAGAAGCCAAC
	Reverse: CAACCACTTCGTGAACAGACAGAAC
SPP1	Forward: GATTGGGACAGCCGTGGGAAG
	Reverse: CATCGGAATGCTCATTGCTCTCATC

#### Statistical analysis

Cells were observed and photographed under a fluorescence microscope. The stained area was quantified using Image J software. Results were shown as mean ± SD and analyzed by one-way analysis of variance (ANOVA). Differences with *P* < 0.05 was considered significant, and statistical tests for each experiment are detailed in the relevant figure legends. Data analysis was performed using GraphPad Prism Software 9.5.

## Results

### Identification of common crosstalk genes in AS and NASH

**Figure 2:**
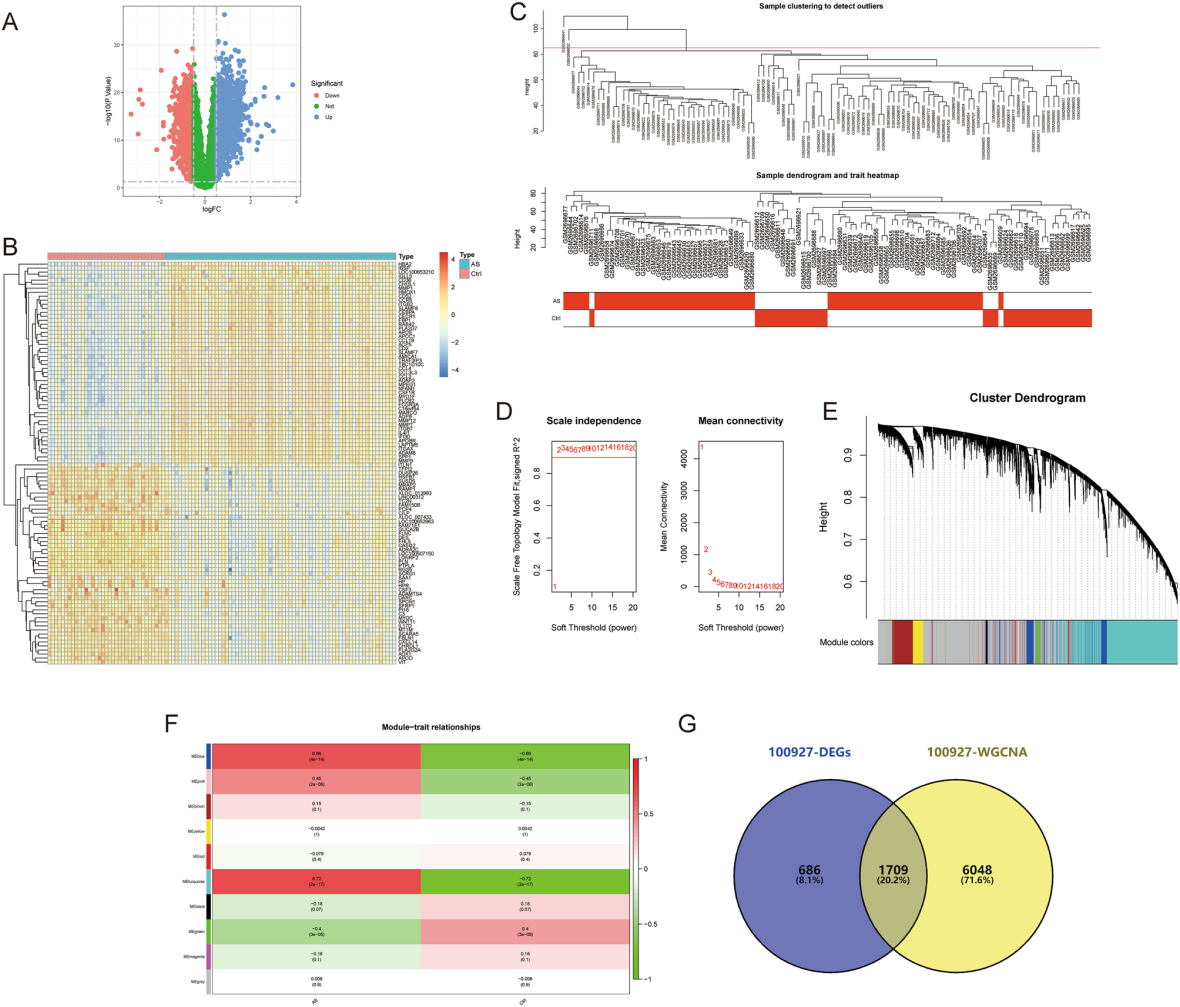
Identification of AS (GSE100927) crosstalk genes by differential expression analysis and WGCNA. (**A**,**B**) Volcano plot (**A**) and heatmap (**B**) for differentially expressed genes (DEGs). (**C**) Sample clustering plot after removing outlier samples. (**D**) Soft-thresholding power analysis was used to obtain the scale-free fit index of network topology. (**E**) Dendrogram of all genes clustered based on the measurement of dissimilarity (1-TOM). (**F**) Heatmap of the correlation between the module eigengenes and clinical traits of AS. (**G**) Venn diagram showing the 1709 crosstalk genes.

**Figure 3:**
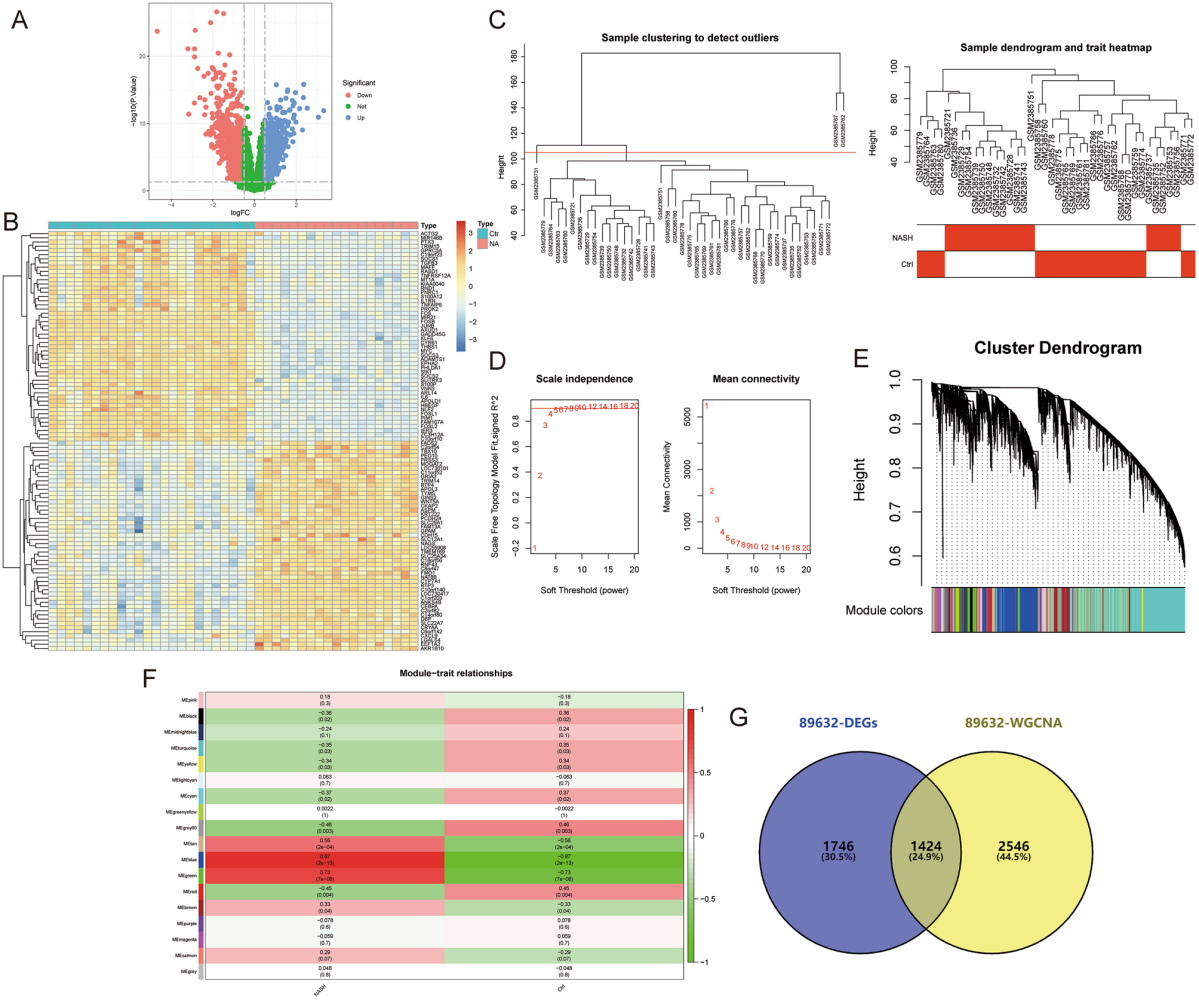
Identification of NASH (GSE89632) crosstalk genes by differential expression analysis and WGCNA. (**A**,**B**) Volcano plot (**A**) and heatmap (**B**) for DEGs. (**C**) Sample clustering plot after removing outlier samples. (**D**) Soft-thresholding power analysis was used to obtain the scale-free fit index of network topology. (**E**) Dendrogram of all genes clustered based on the measurement of dissimilarity (1-TOM). (**F**) Heatmap of the correlation between the module eigengenes and clinical traits of NASH. (**G**) Venn diagram showing the 1424 crosstalk genes.

**Figure 4:**
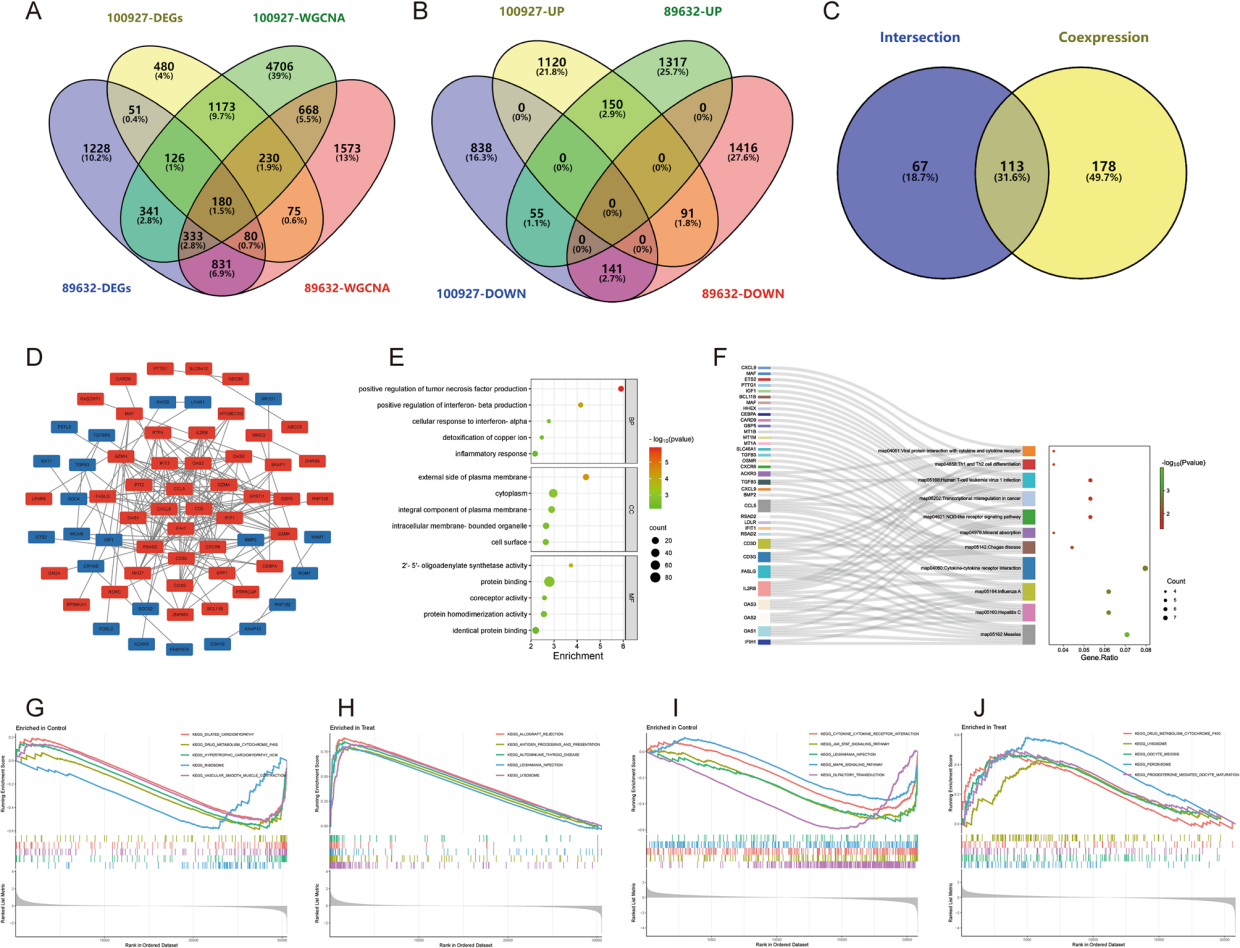
Identification and functional enrichment analysis of AS and NASH common crosstalk genes. (**A**–**C**) Venn diagram showing 113 common crosstalk genes expressed in the same direction. (**D**–**F**) The PPI network (**D**), GO (**E**), and KEGG (**F**) enrichment analysis of common crosstalk genes. (**G**,**H**). The GSEA for normal (**G**) and AS (**H**) samples. (**I**,**J**) The GSEA for normal (**I**) and NASH (**J**) samples.

The data for this study is presented in Supplementary Material B. The differential gene expression analysis for AS-GSE100927 identified 2399 DEGs (Fig. 2A,B). WGCNA identified the “turquoise” module with the highest correlation to AS, containing 7757 genes (Fig. 2C–F). 1709 crosstalk genes for AS were obtained after intersection (Fig. 2G). For NASH-GSE89632, the differential gene expression analysis identified 3170 DEGs (Fig. 3A,B), and WGCNA identified the “blue” module with the highest correlation to NASH, containing 3970 genes (Fig. 3C–F); 1424 crosstalk genes of NASH were obtained after intersection (Fig. 3G). Finally, 180 common crosstalk genes between AS and NASH were identified (Fig. 4A), with 113 expressed in the same direction (Fig. 4B,C).

### Enrichment analysis of common crosstalk genes

GO and KEGG analyses were performed to explore the biological functions and pathways involved. The GO analysis encompassed biological process (BP), cellular component (CC), and molecular function (MF). The 113 common crosstalk genes were imported into the David database for GO and KEGG analysis, of which 57 were correlated with BP, 8 with CC, and 13 with MF, and 11 signaling pathways were obtained by KEGG analysis (*P* < 0.05). The results suggest that the pathogenesis of the co-morbidity pattern of AS and NAHS is related to inflammatory and immune responses, copper ion metabolism, and notably, the NOD-like receptor signaling pathway (Fig. 4E,F).

To determine the potential functions of these genes in GSE100927 and GSE89632, GSEA was used to identify differential regulatory pathways and signaling pathways activated in AS and NASH. The comprehensive analysis indicates that the common pathogenesis of AS and NASH likely involves cardiomyopathy, immunity, metabolism, and cytokine-mediated signaling pathways, such as the JAK-STAT signaling pathway (Fig. 4G–J).

### Construction of PPI network

**Figure 5:**
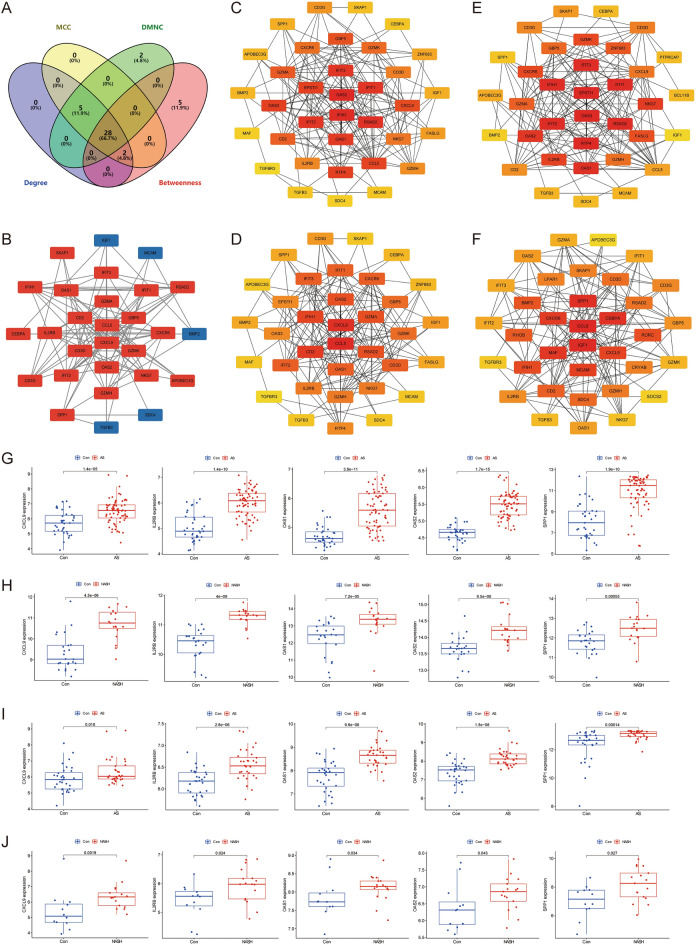
Identification of candidate hub genes. (**A**) Venn diagram showing the 28 candidate hub genes by four algorithms. (**B**) The PPI network of candidate hub genes. (**C**–**F**) The top 35 candidate hub genes obtained by MCC (**C**), DMNC (**D**), Degree (**E**), and Betweenness (**F**). (**G**–**J**) Identification of hub genes in GES100927 (**G**), GSE89632 (**H**), GSE43292 (**I**), and GSE48452 (**J**) single gene differential analysis.

Cytoscape software was utilized to construct a PPI network of 113 common crosstalk genes, which included 66 nodes and 184 edges (Fig. 4D). The top 35 genes ranked by four algorithms (MCC, DMNC, Degree, and Betweenness) were calculated using the “CytoHubba” plugin (Fig. 5C–F). After intersection, 28 candidate Hub genes were obtained (OAS2, IFIH1, RSAD2, IFIT3, IFIT2, IFIT1, OAS1, CCL5, CXCL9, GBP5, GZMA, CD2, GZMK, CXCR6, NKG7, CD3D, IL2RB, GZMH, CD3G, SPP1, IGF1, TGFB3, APOBEC3G, BMP2, SKAP1, MCAM, CEBPA, and SDC4) (Fig. 5A) (Table 2), and a candidate Hub gene PPI network with 28 nodes and 115 edges was constructed (Fig. 5B).

**Table 2:** Screening of candidate Hub genes.

No.	Gene	MCC	DMNC	Degree	Betweenness
1	OAS2	443,526	1.482	26	46.364
2	IFIH1	443,522	1.639	28	245.55
3	RSAD2	443,521	1.639	26	141.55
4	IFIT1	443,520	1.639	24	35.551
5	IFIT2	443,520	1.639	24	35.551
6	IFIT3	443,520	1.639	24	35.551
7	OAS1	403,206	1.493	24	42.713
8	CCL5	92,462	0.833	46	1325.690
9	CXCL9	81,505	1.122	32	253.558
10	GBP5	40,368	1.222	22	46.551
11	GZMA	11,928	1.277	26	33.341
12	CD2	11,818	1.001	30	194.249
13	GZMK	11,640	1.391	22	20.666
14	CXCR6	11,161	1.391	24	309.498
15	NKG7	10,920	1.437	20	15.104
16	CD3D	10,810	1.054	24	164.226
17	IL2RB	5881	1.432	20	53.121
18	GZMH	5186	1.157	20	108.649
19	CD3G	728	1.050	16	74.851
20	IGF1	12	0.475	16	568.033
21	SPP1	12	0.571	12	393.133
22	BMP2	8	0.568	12	220.019
23	APOBEC3G	8	0.758	8	5.159
24	SKAP1	7	0.927	8	106.000
25	CEBPA	5	0.616	10	378.395
26	MCAM	5	0.618	8	307.000
27	TGFB3	5	0.618	8	19.619
28	SDC4	4	0.616	8	171.762

### Screening of Hub genes

**Figure 6:**
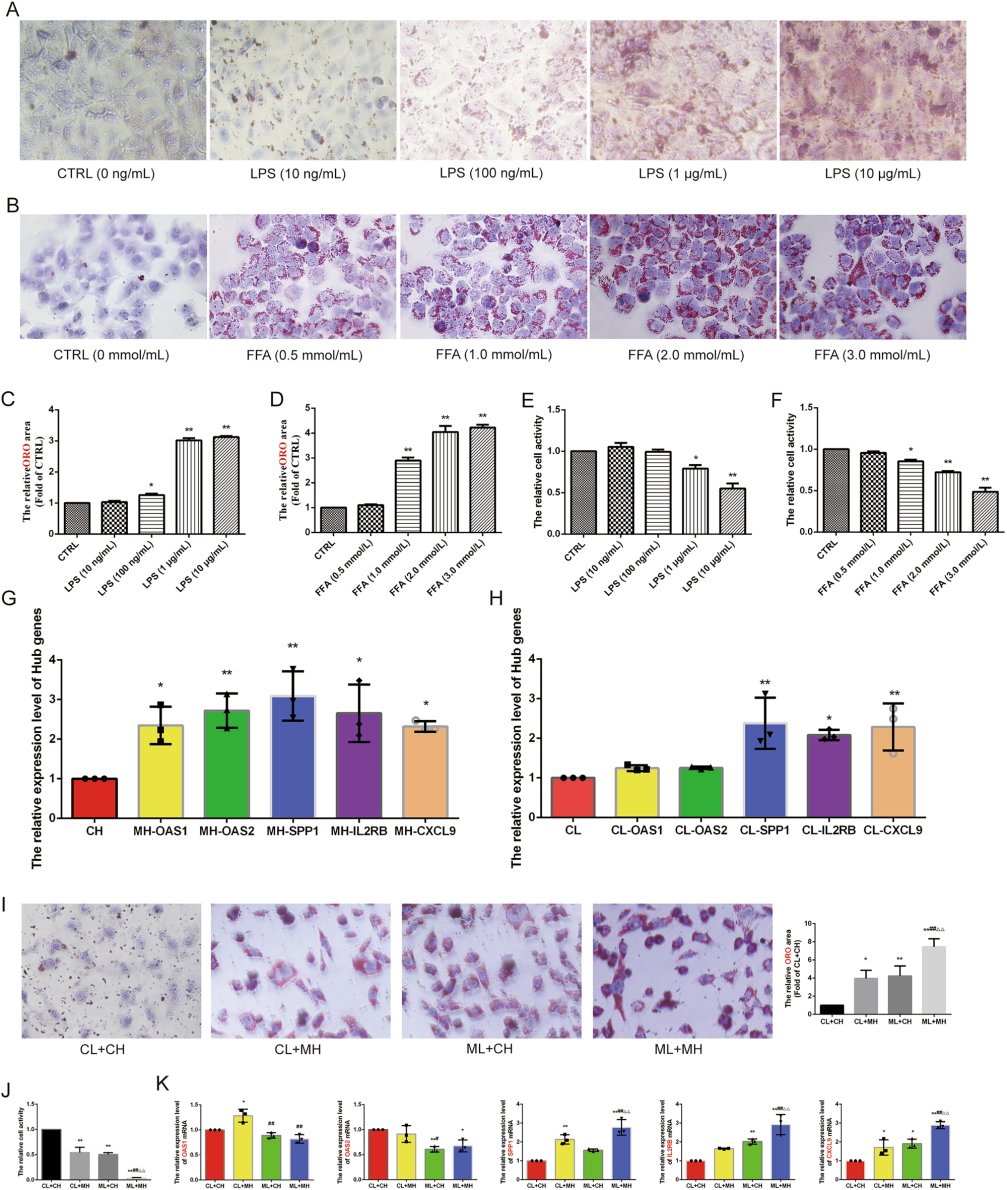
Experimental validation and screening of Hub genes. (**A**) The ORO staining of HUVECs was induced by different concentrations of LPS to construct the AS model (*n* = 5, bar = 500 μm). (**B**) The ORO staining of L02 cells was induced by different concentrations of FFA to construct the NASH model (*n* = 5, bar = 500 μm). (**C**,**D**) The relative cell area of ORO staining for the AS model (**C**) and the NASH model (**D**). (**E**) CCK8 to detect the relative cell viability of HUVECs induced by different concentrations of LPS. (**F**) CCK8 to detect the relative cell viability of L02 cells induced by different concentrations of FFA. (**G**,**H**) qRT-PCR to detect the relative expression levels of Hub genes in the AS model (**G**) and the NASH model (H) (*n* = 3). (**I**) The ORO staining of the co-morbidity model. (**J**) The relative cell viability of the co-morbidity model (*n* = 5, bar = 500 μm). (**K**) qRT-PCR to detect the relative expression levels of Hub genes in the co-morbidity model (*n* = 3). *P < 0.05 vs the first group, **P < 0.01 vs the first group, ^#^*P* < 0.05 vs the CL + MH group, ^##^*P* < 0.01 vs the CL + MH group, ^△^*P* < 0.05 vs the ML + CH group, ^△△^*P* < 0.01 vs the ML + CH group.

Validation using the GSE43292 and GSE48452 datasets revealed that five genes were significantly upregulated (CXCL9, IL2RB, OAS1, OAS2, and SPP1) in both AS and NASH groups compared to the control group (Fig. 5G–J). In vitro experiments further screened these Hub genes using an AS model induced by LPS (1.0 μg/mL) in HUVECs and a NASH model induced by FFA (2.0 mmol/L) (oleic acid = 2 mmol/L, palmitic acid = 1 mmol/L) in L02 cells. These models exhibited significant lipid accumulation and reduced cell activity (Fig. 6A–F). qRT-PCR results showed that CXCL9 (*P* < 0.05), IL2RB (*P* < 0.05), OAS1 (*P* < 0.05), OAS2 (*P* < 0.01), and SPP1 (*P* < 0.01) were significantly up-regulated in the AS model, while CXCL9 (*P* < 0.01), IL2RB (*P* < 0.05), and SPP1 (*P* < 0.01) were significantly up-regulated in the NASH model (Fig. 6G,H). HUVECs and L02 cells co-culture co-morbidity model showed more lipid accumulation and lower cell activity (Fig. 6I,J). Compared with the CL + CH group, the ML + MH group had lower expression levels of OAS2 (*P* < 0.05) and higher expression levels of SPP1 (*P* < 0.01), IL2RB (*P* < 0.01), and CXCL9 (*P* < 0.01). Compared with the CL + MH and ML + CH groups, the ML + MH group had higher expression levels of SPP1 (*P* < 0.01) (*P* < 0.01), IL2RB (*P* < 0.01) (*P* < 0.01), and CXCL9 (*P* < 0.01) (*P* < 0.01) (Fig. 6K). Therefore, CXCL9, IL2RB, and SPP1 were identified as hub genes.

### Evaluation of the diagnostic efficacy of Hub genes

**Figure 7:**
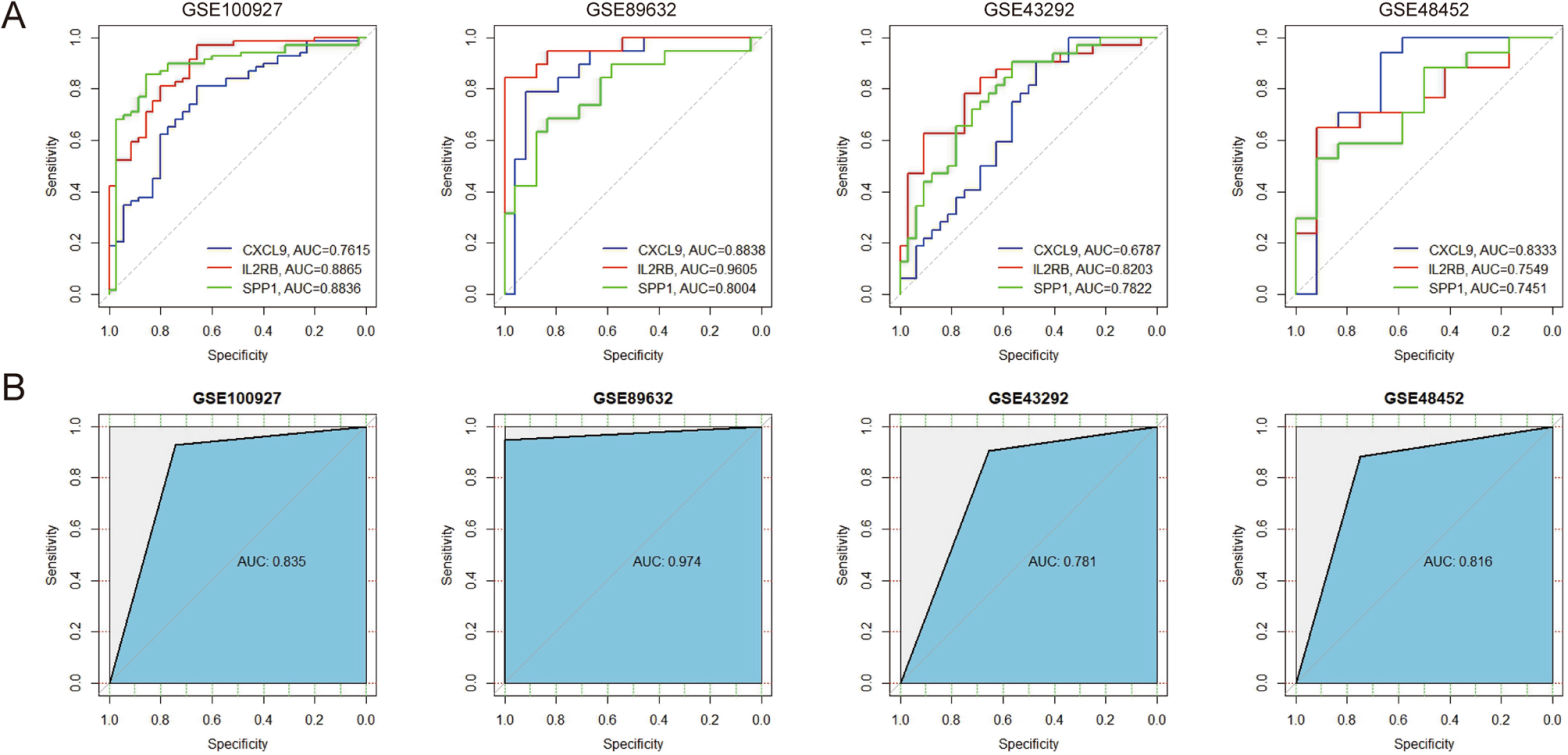
Receiver operating characteristic (ROC) curve and support vector machine (SVM) models to validate the diagnostic efficacy of hub genes in AS and NASH. (**A**) ROC curve to validate the diagnostic efficacy of individual hub genes. (**B**) SVM model to validate the efficacy of combined hub gene diagnosis. The X-axis represents specificity, and the Y-axis represents sensitivity, AUC stands for “rea under the curve”.

ROC curves for these Hub genes across four datasets were generated using the “pROC” R package, and the area under curve (AUC) values for these Hub genes exceeded 0.6, indicating that the diagnostic efficacy of these Hub genes was excellent (Fig. 7A). SVM models were developed for these genes showed AUC values above 0.75, indicating satisfactory combined diagnostic efficacy (Fig. 7B).

### Immune infiltration analysis

**Figure 8:**
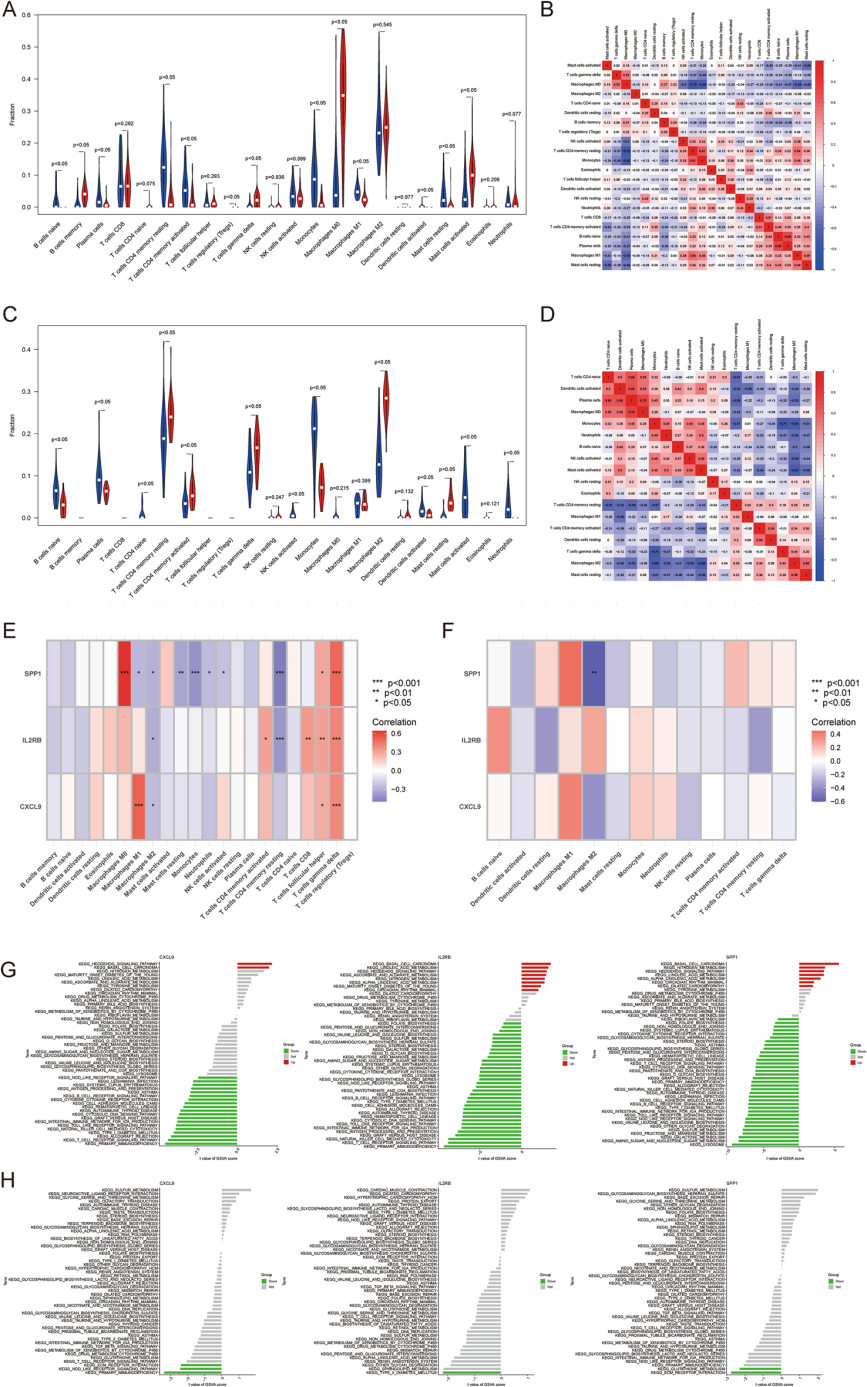
Immune infiltration analysis and GSVA. (**A**,**C**). Differences in immune infiltration between AS (**A**) and NASH (**C**) and control samples. (**B**,**D**) Correlation between 22 immune cells in AS (**B**) and NASH (D). (**E**,**F**) Correlation between hub genes and immune cells in AS (**E**) and NASH (**F**). (**G**,**H**) GSVA analysis of hub genes in AS (**G**) and NASH (**H**).

To further reveal the immune microenvironment in AS and NASH, specific immune cell types infiltrating into AS and NASH tissues were analyzed using the CIBERSORT method. Among the 22 immune cells studied, the results showed significantly higher levels of memory B cells, γ–δ T cells, M0 macrophages, and activated mast cells in AS (*P* < 0.05) (Fig. 8A), and further analysis of CIBERSORT scores showed a strong positive correlation of plasma cells with naive B cells, monocytes, and resting memory CD4 T cells, while a strong negative correlation of M0 macrophages with resting memory CD4 T cells and monocytes (Fig. 8B). The levels of resting memory CD4 T cells, activated memory CD4 T cells, γ–δ T cells, M2 macrophages, and resting mast cells were significantly higher in NASH (*P* < 0.05) (Fig. 8C) and further analysis of CIBERSORT scores showed a strong positive correlation between plasma cells and M0 macrophages, while a strong negative correlation was found between γ–δ T cells and monocytes (Fig. 8D).

In addition, correlation analyses showed that multiple immune cells were significantly associated with five Hub genes, particularly M0 macrophages, resting memory CD4 T cells, follicular helper T cells, γ–δ T cells in AS, and M2 macrophages in NASH (Fig. 8E,F).

### GEVA

To further explore the potential mechanisms of Hub genes in AS and NASH, differences in the enrichment of each Hub gene pathway were analyzed using GSVA. The results showed that CXCL9, IL2RB, and SPP1 were mainly down-regulated in AS and NASH in immune and metabolism-related pathways, especially the metabolism of various fatty acids, the Toll-like receptor signaling pathway, the T cell receptor signaling pathway, and the NOD-like receptor signaling pathway, and up-regulated in some cancer and biosynthesis related pathways, especially basal cell carcinoma, steroid biosynthesis, terpenoid backbone biosynthesis, nitrogen metabolism, sulfur metabolism, Linoleic acid metabolism, and the hedgehog signaling pathway (Fig. 8G,H).

### Hub gene–drug interaction

Importing the three Hub genes into the DGIbd database yielded 28 potential drugs, of which 11 were FDA-approved, while no FDA-approved drugs were available for CXCL9. The drugs corresponding to IL2RB are aldesleukin, denileukin diftitox, daclizumab, and basiliximab; and the drugs corresponding to SPP1 are calcitonin, anhydrous tacrolimus, tretinoin, chondroitin sulfates, zoledronic acid anhydrous, pamidronate, and gentamicin. Finally, five potential drugs were screened by literature search for calcitonin, anhydrous tacrolimus, tretinoin, chondroitin sulfates, and zoledronic acid anhydrous.

### Construction of common miRNA–mRNA network

There were 30 potential miRNAs for CXCL9, 40 for IL2RB, and 6 for SPP1 obtained by database prediction, of which has-miR-185 regulated CXCL9 and IL2RB, and hsa-miR-335 regulated CXCL9 and SPP1.

## Discussion

Several studies have found that cardiovascular risk increases with the severity of fatty liver injury and the degree of fibrosis^35,36^. Furthermore, AS and NASH share many common risk factors, such as obesity, diabetes, hypertension, and dyslipidemia. This suggests that there is an identical pathogenesis between AS and NASH. In the present study, it was found that their shared pathogenesis is associated with biological processes including inflammatory response, immune response, glycolipid metabolism, and copper ion metabolism, which may act through the NOD-like receptor signaling pathway and the JAK-STAT signaling pathway (Fig. 4E–J). The role of inflammation in the co-morbidity pattern of AS and NASH has been validated. NASH is due to chronic inflammatory infiltration of hepatocytes, and AS is due to chronic inflammatory infiltration of the vascular wall. Hepatic secretion of inflammatory molecules, considered pro-atherogenic, may contribute to vascular endothelial dysfunction, resulting in atherosclerosis and CVDs^37^.

The inflammatory response plays a critical role in the co-morbidity mechanism of AS and NASH. Chronic inflammatory responses in the vascular wall led to endothelial cell damage, vessel wall thickening, and stiffening, which can result in AS formation and plaque development. Lipid accumulation results in prolonged inflammatory infiltration of the liver, leading to the development of hepatic fibrosis. In addition, pro-inflammatory cytokines and chemokines are secreted during liver inflammation, which may lead to vascular endothelial dysfunction and increase the risk of AS and CVDs^37^.

The previous immune infiltration analysis revealed disturbances in the immune microenvironment of AS and NASH, particularly involving γ–δ T cells and macrophages. γ–δ T cells primarily reside in epithelial and mucosal layers and play a role in defense against infection by producing cytokines such as IFN-γ, TNF-α, IL-17, and Th2 cytokines^38,39^. The absence of γ–δ T cells, which are the most abundant T cells in AS lesions, reduced neutrophil infiltration and delayed AS lesions development^40–43^. γ–δ T cells constitute 3–5% of adult lymphocytes in the liver, a proportion much higher than other tissues^44^. An increased in γ–δ T17 cells occurs in liver inflammation and fibrosis. Hepatic γ–δ T cells produce IL-17A, which accelerates the development of NAFLD^45,46^. Mice fed a high-fat diet showed increased hepatic γ–δ T17 cells producing IL-17A, leading to steatohepatitis, liver damage, and impaired glucose metabolism^47^. Strategies such as deleting, depleting, or interrupting the recruitment of γ–δ T cells have prevented diet-induced steatohepatitis and accelerated resolution^48^. Hepatic γ–δ T cells also contribute to NASH by bridging innate and adaptive immunity^49,50^. Thus, the highly expressed γ–δ T cells are a common factor in the pathogenesis of both AS and NASH.

Macrophage infiltration plays a central role in AS and was previously thought to be a late consequence of steatosis. However, recent studies have shown that the activation and infiltration of macrophages are also early initiating event in NASH. Inhibition of macrophage infiltration has been shown to reduce the development of hepatic inflammation and AS, highlighting it as one of the common mechanisms linking AS and NASH^51,52^.

Copper is an essential trace element involved in oxidative stress and energy metabolism. It has been found that copper levels are closely related to the morbidity and mortality of NASH and atherosclerotic CVDs; thus, abnormal copper metabolism may be one of the mechanisms causing the common pathogenesis of AS and NASH^53,54^. High serum copper levels accelerate the formation of atherosclerotic plaque by affecting lipid metabolism, oxidative stress, low-density lipoprotein oxidation, and inflammation, thus increasing the risk of atherosclerotic heart disease^55,56^. In addition, copper ion-oxidized low-density lipoprotein generates intracellular oxidative stress through lipid peroxidation products, induces Tyr phosphorylation and activation of JAK2, STAT1, and STAT3, and participates in the JAK/STAT pathway, which promotes the development of AS and NASH^57^.

In this study, 28 candidate Hub genes were first obtained after screening for differentially expressed genes between normal individuals and those with AS and NASH. Then, three Hub genes (CXCL9, IL2RB, and SPP1) were confirmed through an external validation set and qRT-PCR verification. The ROC curves and SVM models were used to verify the validity of the diagnostic efficacy. Furthermore, exploring the biological properties of these three Hub genes also explores the common pathogenesis of AS and NASH, potentially offering a new strategy for the early diagnosis of these conditions. SVM is a supervised machine learning algorithm used for binary classification prediction and regression-based attribute value prediction. It is capable of handling high-dimensional data, has strong generalization ability, and is widely used in disease gene screening and drug development. However, SVM has drawbacks, such as its sensitivity to noisy data and its primary applicability to binary classification problems. Future research may focus on constructing multiple models, selecting better and more appropriate models for screening, and improving overall model performance^58–60^.

CXCL9, also known as monokine induced by interferon-γ, plays a role in immune cell migration and inflammatory responses. It is highly expressed in patients with AS and is associated with carotid intima-media thickness and a coronary artery calcification score^61–63^. It may contribute to T lymphocyte recruitment and proliferation at sites of inflammation^64,65^. CXCL9 expression is also elevated in NAFLD patients and a mouse model of NASH, with the CXCL9 protein detected in hepatocytes and sinusoidal endothelium in areas infiltrated by inflammatory cells^66,67^. Recent studies suggest that CXCL9 promotes NASH development and progression by activating the p-JNK pathway, promoting Th17 cell proliferation, and disrupting Treg/Th17 cell homeostasis in a mouse model of NASH^68^. Bioinformatics analyses have consistently shown CXCL9 upregulation in human and animal models of NAFLD and NASH, supporting the findings of the present study^69,70^. Overall, CXCL9 is recognized as a potential biomarker for AS and NASH, with its elevated mRNA levels potentially indicating early development of both conditions.

IL2RB is a key constituent subunit of the IL-2 cytokine receptor, which plays a central role in IL-2 signaling and is also implicated in the development and progression of AS and NASH^71^. Activation of IL2RB regulates the proliferation and differentiation of immune cells such as Treg and NK cells^72^. Treg cells produce high levels of the IL-10 inflammation suppressor to inhibit inflammation, as well as high levels of TGF-β, which regulate macrophage polarization and stabilize AS plaques^73,74^. However, Treg cells, which are highly expressed in NASH, can accelerate hepatic steatosis during the development of NAFLD and NASH and do not act as modulators of metabolic inflammation but rather enhance it^75,76^. IL2RB also supports the differentiation of T-lymphocytes towards Th1 and promotes the production of the pro-inflammatory factor interferon-γ, affecting the progression of AS by modulating the inflammatory response^77,78^. IL-2Rβγ signaling in lymphocytes promotes systemic inflammation and reduces plasma cholesterol in atherosclerotic mice^79^. Construction of a NASH model in Parma minipigs, continuously fed a high-fat and high-sucrose diet for 23 months, reveals high expression of IL2RB as an inflammatory gene^80^.

SPP1, also known as osteopontin (OPN), is involved in the inflammatory milieu of AS and is highly expressed in endothelial cells, macrophages, and vascular smooth muscle cells within AS plaques^81,82^. It is also elevated in AS patients and correlates with the occurrence and severity of coronary artery disease, serving as a marker for monitoring coronary AS severity and predicting cardiovascular event mortality^83,84^. Overexpression of OPN significantly promotes fatty streak formation in AS mice and inhibits IL-10 production by macrophages^85^. OPN also increases autophagosome formation, autophagy-related gene expression, and cell death associated with AS development^86^. SPP1 is closely associated with the development, progression, and prognosis of fatty liver, liver fibrosis, and hepatocellular carcinoma^87^. Its expressions are significantly elevated in the plasma of NASH patients and correlate with histological features of NASH severity^88,89^. SPP1 promotes inflammatory responses, cellular activation, proliferation, and migration, and inhibits autophagy in liver disorders. In a porcine model of NASH, SPP1 expression was positively correlated with lipid droplet area and inflammation but decreased when NASH was reversed^90^. However, a lack of SPP1 exacerbated lipid accumulation, hepatocyte apoptosis, and fibrosis, indicating a complex role in NASH pathogenesis^91^.

Statins are the primary drugs for preventing cardiovascular disease and constitute the primary class of lipid-lowering drugs for treating AS and NASH. Studies have found that the use of statins significantly reduces the levels of CXCL9, IL2RB, and SPP1, suggesting that statins may exert their effects by targeting these proteins^62,92–96^.

Finally, the Hub gene was imported into the DGIbd database to obtain 11 FDA-approved drugs, which were further screened through a literature search to find five potential drugs capable of treating AS and NASH, namely, calcitonin, anhydrous tacrolimus, tretinoin, chondroitin sulfates, and zoledronic acid anhydrous. Except for calcitonin, which down-regulated the expression of SPP1, all the identified drugs up-regulated the expression of SPP1/OPN^97–102^.

Calcitonin has been used to prevent experimental immunarteriosclerosis^103^, which may modulate vascular homeostasis by decreasing lipid levels and adipogenesis in cells^104^, preventing the increase of calcium influx, and enhancing the permeability of the arterial smooth muscle^105^. In addition, calcitonin extracted from salmon is more effective in inhibiting gastric emptying, promoting gallbladder relaxation, increasing energy expenditure, and inducing satiety and weight loss. This demonstrates its great therapeutic potential in obesity and NAFLD^106^.

Tacrolimus reduced AS formation by inhibiting ROS in macrophages and activating the NLRP3 inflammasome, as well as decreasing IL-1β and IL-18 release^107^. In addition, tacrolimus-eluting stents inhibited neointimal hyperplasia after stent placement via the calcineurin/NFAT/IL-2 signaling pathway^108^. Tacrolimus also reduced hepatic lipid accumulation and improved the lipid profile in fast-food diet mice by inhibiting the expression of adipogenic factors such as PPAR-γ, SREBP-1, and SCD-1. It also reduced apoptosis and suppressed the level of hepatic fibrosis^109^.

Tretinoin plays a potential role in the prevention of CVDs, and studies have shown that it can influence the development of AS by regulating various biological processes in immune cells, such as cell proliferation, migration, and phenotypic transformation. It is also involved in the regulation of blood glucose concentration, lipid metabolism, and the inflammatory response, thereby improving AS^110^. In a study, serum tretinoin concentrations were found to be significantly lower in NASH patients than in healthy subjects^111^. Tretinoin supplementation significantly improved hepatic steatosis, glucose tolerance, and insulin sensitivity in mice induced by a high-fat diet^112^.

Chondroitin sulfates (CS) are a class of drugs recommended for the treatment of osteoarthritis, and studies have found that osteoarthritis patients treated with high doses of CS are less likely to develop coronary artery disease^113,114^. Furthermore, patients with AS have been observed to have lower mortality rates, suggesting that CS may have beneficial cardiovascular effects^115^. Several studies have suggested that CS may delay the progression of AS lesions by inhibiting inflammation and foam cell formation. This is achieved by decreasing the expression of pro-inflammatory factors (e.g., CRP, IL-6, COX-2, MCP1, TNF-α, and IL-1β), cytokines (e.g., CCL2), and other cell adhesion molecules^116–118^. In addition, CS supplementation was found to improve insulin resistance, liver function, and lipid levels in NAFLD mice by regulating the composition of the intestinal flora^119^.

Recent clinical studies have shown that zoledronic acid improves the lipid profile of patients, delays the progression of vascular calcification and AS, reduces the risk of cardiovascular disease, and decreases mortality^120,121^. It may affect the development of AS and NASH by inhibiting the proliferation, adhesion, and migration of vascular smooth muscle cells^122^, blocking the mevalonate pathway, decreasing hepatic TNF-α and VEGF expression, regulating cholesterol synthesis, and improving the lipid profile. Zoledronic acid also enhances hepatic function and prevents portal hypertension^123,124^.

MiRNAs, non-coding RNAs of 21–24 nucleotides in length, regulate gene expression by promoting or inhibiting mRNA degradation and translation. miRNAs have been shown to be implicated in the biological processes and pathogenesis of AS and NASH. Several studies have summarized the roles of miRNAs in AS and NASH, and this study, based on multiple miRNA research tools, identified has-miR-185 and hsa-miR-335 as potentially playing significant roles in AS and NASH^125,126^. It was found that miR-185 could delay the development of AS and NAFLD/NASH by regulating various biological processes, including cell proliferation, migration, and invasion^127^; enhancing lipid metabolism^128,129^; inhibiting inflammation and apoptosis^130^; and affecting insulin resistance^131^. miR-335 has been shown to inhibit the intrinsic immune response of macrophages, reducing the formation of vulnerable plaques in AS^132^; improving endothelial function^133^; and regulating the proliferation and phenotypic switching of smooth muscle cells^134^, thereby slowing the progression of AS. Additionally, miR-335 influences NAFLD and NASH through the modulation of insulin resistance and has been recognized as a potential diagnostic biomarker for NAFLD^135–137^.

To summarize, this study leverages bioinformatics to identify potential common biomarkers and explore the pathogenesis of AS and NASH. Utilizing multiple topological algorithms, the research underwent external and in vitro experimental validation to screen for hub genes. Subsequently, the diagnostic efficacy of these genes was assessed using SVM modeling and ROC curves. The study further conducted GSVA and immune-infiltration analyses on the identified hub genes. This research is innovative as it explores the connection between AS and NASH, which are linked by similar risk factors and share common pathogenic mechanisms. By employing a co-morbidity pattern, the study utilizes bioinformatics analysis to investigate the interplay between AS and NASH, with a focus on mining potential biomarkers and understanding the shared pathogenesis. This approach, which has not been extensively explored by scholars, offers new insights that can inform the investigation of chronic diseases and patterns of co-morbidity. Then this study constructed an AS foam cell model, a NASH hepatocyte steatosis model, and a co-morbid in vitro model of AS and NASH to verify the expression levels of candidate hub genes. It culminated in the identification of potential drugs for the treatment of both AS and NASH based on these hub genes, which holds significant implications for clinical guidance. This research is both promising and innovative, delving into the investigation of biomarkers and pathogenesis within the context of co-morbid AS and NASH. The efficacy of the hub genes was validated using SVM modeling, a method that adds rigor to our findings. Additionally, the study offers a foundation for other scholars to explore the complexities of co-morbid conditions.

However, this study still has some limitations. Firstly, for the dual-disease bioinformatics analysis, the database did not contain data from patients with AS and NASH co-morbidities, which may result in the absence of some potential diagnostic genes. Secondly, this study was validated using human cell lines for in vitro experiments, and a co-culture co-morbidity model was constructed for further validation, but it lacked further validation in animal and human tissues. In addition, this study used SVM models for Hub gene screening, and constructing multiple models and then screening them appeared to have different results. Lastly, although the literature search identified five potential therapeutic drugs and two related miRNAs, their specific mechanisms and efficacy need to be further investigated.

## Conclusions

AS and NASH share several common pathogenetic mechanisms related to inflammatory response, immune response, glycolipid metabolism, and copper ion metabolism. Three hub genes (CXCL9, IL2RB, and SPP1) serve as potential biomarkers for the diagnosis of the comorbidities of AS and NASH, either independently or in combination, with a certain degree of accuracy and specificity. Five potential drugs for the treatment of AS and NASH, namely, calcitonin, anhydrous tacrolimus, tretinoin, chondroitin sulfates, and zoledronic acid anhydrous.

### Supplementary Information

Supplementary Information 1.

Supplementary Information 2.

## Data Availability

The following information was supplied regarding data availability: Data is available at Supplementary Material A, B and NCBI GEO: GSE100927, GSE89632, GES43292, and GSE48452. Additionally, any analytic technology-related questions can be directly contacted by the corresponding author.

## Abbreviations

AS: Atherosclerosis
AUC: Area under curve
BP: Biological process
CC: Cellular component
CS: Chondroitin sulfates
CVDs: Cardiovascular diseases
DEGs: Differentially expressed genes
FDA: Food and Drug Administration
FFA: Free fatty acid
GEO: Gene Expression Omnibus
GO: Gene Ontology
GSEA: Gene set enrichment analysis
GSVA: Gene set variation analysis
HUVECs: Human umbilical vein endothelial cells
KEGG: Kyoto Encyclopedia of Genes and Genomes
LPS: Lipopolysaccharide
MF: Molecular function
NAFLD: Nonalcoholic fatty liver disease
NASH: Non-alcoholic steatohepatitis
OPN: Osteopontin
ORO: Oil Red O
PPI: Protein–protein interaction
ROC: Receiver operating characteristic
qRT-PCR: Quantitative real-time polymerase chain reaction
SVM: Support vector machine
WGCNA: Weighted gene co-expression network analysis
